# Investigation of Effect of Part-Build Directions and Build Orientations on Tension–Tension Mode Fatigue Behavior of Acrylonitrile Butadiene Styrene Material Printed Using Fused Filament Fabrication Technology

**DOI:** 10.3390/ma17205133

**Published:** 2024-10-21

**Authors:** Ibrahim S. El-Deeb, Cezary Grabowik, Ehssan Esmael, Ahmed Nabhan, Maher Rashad, Saad Ebied

**Affiliations:** 1 Department of Production Engineering and Mechanical Design, Faculty of Engineering, Tanta University, Sibirbay Campus, Tanta 31527, Egypt; ehssan152151@f-eng.tanta.edu.eg (E.E.); dr.maherrashad@f-eng.tanta.edu.eg (M.R.); saad_ebied@f-eng.tanta.edu.eg (S.E.); 2 Department of Engineering Processes Automation and Integrated Manufacturing Systems, Silesian University of Technology, Konarskiego 18A Str., 44-100 Gliwice, Poland; 3 Department of Production Engineering and Mechanical Design, Faculty of Engineering, Minia University, Minya 61111, Egypt; a.nabhan@minia.edu.eg

**Keywords:** additive manufacturing, fused filament fabrication (FFF), acrylonitrile butadiene styrene (ABS), building orientation, building angles, tensile fatigue

## Abstract

This article explores the fatigue characteristics of acrylonitrile butadiene styrene (ABS) components fabricated using fused filament fabrication (FFF) additive manufacturing technology. ABS is frequently used as a polymeric thermoplastic material in open-source FFF machines for a variety of engineering applications. However, a comprehensive understanding of the mechanical properties and execution of FFF-processed ABS components is necessary. Currently, there is limited knowledge regarding the fatigue behavior of ABS components manufactured using FFF AM technology. The primary target of this study is to evaluate the results of part-build directions and build orientation angles on the tensile fatigue behavior exhibited by ABS material. To obtain this target, an empirical investigation was carried out to assess the influence of building angles and orientation on the fatigue characteristics of ABS components produced using FFF. The test samples were printed in three distinct directions, including Upright, On Edge, and Flat, and with varying orientation angles ([0°, 90°], [15°, 75°], [30°, 60°], [45°]), using a 50% filling density. The empirical data suggest that, at each printing angle, the On-Edge building orientation sample exhibited the most prolonged vibrational duration before fracturing. In this investigation, we found that the On-Edge printing direction significantly outperformed the other orientations in fatigue life under cyclic loading with 1592 loading cycles when printed with an orientation angle of 15°–75°. The number of loading cycles was 290 and 39 when printed with the same orientation angle for the Flat and Upright printing directions, respectively. This result underscores the importance of orientation in the mechanical performance of FFF-manufactured ABS materials. These findings enhance our comprehension of the influence exerted by building orientation and building angles on the fatigue properties of FFF-produced test samples. Moreover, the research outcomes supply informative perspectives on the selection of building direction and building orientation angles for the design of 3D-printed thermoplastic components intended for fatigue cyclic-loading applications.

## 1. Introduction

Various additive manufacturing (AM) techniques have been employed to produce rapid and accurate prototypes for the purpose of validating designs. Additive manufacturing (AM) encompasses a diverse range of techniques that fabricate objects layer by layer from digital models, offering numerous advantages such as design flexibility, rapid prototyping, and customization. One of the most widely used AM methods is Fused Deposition Modeling (FDM), which extrudes thermoplastic filaments to build up layers. Stereolithography (SLA) employs a UV laser to selectively cure liquid resin into solid layers, offering high precision and smooth surface finishes. Selective Laser Sintering (SLS) uses a laser to sinter powdered materials, such as polymers or metals, into solid layers, enabling the fabrication of complex geometries and functional prototypes. Electron Beam Melting (EBM) and Direct Metal Laser Sintering (DMLS) are metal AM processes that use high-energy beams to melt and fuse metal powders, producing parts with high strength and density suitable for aerospace and medical applications. Binder Jetting (BJ) selectively deposits binding agents onto powder beds, bonding them together layer by layer, and is often used for producing sand molds, ceramic parts, and metal components. Multi Jet Fusion (MJF) utilizes inkjet arrays to selectively fuse powder materials with a binding agent and energy source, enabling the fast and efficient production of functional parts. These AM methods offer a wide range of capabilities and applications across industries, including aerospace, automotive, medical, and consumer goods [1,2,3]. Due to its accessibility, speed of production, and adaptability, FFF, a material-extrusion additive process, has emerged as one of the most popular used techniques for AM, owing to its accessibility, speed of production, and adaptability. Furthermore, due to its affordability, rapid production rate, and adaptability, FFF represents one of the most effective AM techniques available. This novel technique, which is named AM, enables the direct production of physical objects from three-dimensional computer-aided design (CAD) models, eliminating the need for conventional tools or complex programming. AM is a collection of technologies that utilize three-dimensional model data to construct objects layer by layer [4]. This technology has demonstrated promising potential as well as alternate methods for processing materials for application in many different industries [4,5]. Additive manufacturing (AM) offers greater design flexibility, allowing businesses to rapidly and efficiently transform initial design concepts into functional prototypes and final products. Creating a 3D model of the desired object is strongly recommended. FFF is one of the most extensively utilized AM procedures in customized and small-scale manufacturing industries due to its safe, rapid, and efficient operation, modification flexibility, and economical process [6]. In addition to functional prototyping, FFF has also found application in the aerospace, automotive, medical, and biomechanical industries [7,8]. Despite the many advantages of FFF, relatively little is known about the fatigue performance of thermoplastic materials produced using this technology.

The additive manufacturing, particularly fused filament fabrication (FFF), of thermoplastic composites finds diverse applications across industries. In aerospace, for example, this technology can be used for manufacturing drone frames, as indicated in the following reference [9]. Also, it is used to manufacture lightweight and durable components like cabin brackets and air ducts using carbon fiber-reinforced thermoplastics [10]. Automotive industries employ this technology for producing interior trim panels and exterior body panels using glass fiber-reinforced nylon, enhancing strength, and reducing weight [11]. In the medical field, FFF is utilized for fabricating patient-specific orthopedic implants such as bone plates and prosthetic limbs using biocompatible polymer composites [12,13]. Similarly, in the biomedical sector, customized surgical instruments and dental devices like anatomical models and dental crowns are manufactured using reinforced thermoplastic polymers [14,15]. These examples illustrate the versatility of FFF technology in creating complex, functional, and customized parts across aerospace, automotive, medical, and biomedical sectors.

Additive manufacturing (AM) has largely supplanted rapid prototyping as the primary application of three-dimensional (3D) printing, owing to its ability to produce complex geometries with minimal setup and operator intervention [16,17,18]. Assessing the mechanical performance of 3D-printed thermoplastic components is crucial for their application in end-use products. While metals have historically dominated the AM market for final parts, there is a growing trend towards the use of polymers [16,18]. The mechanical properties of FFF-produced thermoplastics have been well-characterized [19], but relatively little is known about their fatigue performance. Therefore, investigating thermoplastic AM components is important to fully understand their fatigue performance.

FFF’s accessibility and affordability have contributed to its popularity in additive manufacturing [20]. The process involves melting a thermoplastic filament in a heated nozzle and depositing it in successive layers on a build bed to construct the desired 3D object [21,22]. El-Deeb et al., presented a novel and extremely low-cost approach of reverse engineering for reconstructing a CAD model of the physical object [23]. Many scanning systems were developed in the last decades and used for building up 3D models [24]. These cost-effective AM machines are useful not only for communication and learning in education [25] but also prove useful for validating designs and engineering functional testing machine components. One such example is the Cube 3D Printers, which are compact, economical machines that enable rapid printing of ABS and polylactic acid (PLA) materials [26,27].

Various thermoplastics and composites are now utilized to successfully build 3D objects using the FFF technology [28,29,30]. The low cost, lightweight nature, and ability to fabricate intricate geometries have made FFF-processed thermoplastic components popular in both engineering and medical sectors [31]. While thermoplastics are frequently used in load-bearing engineering applications, they are more commonly employed in biomedical and tissue engineering—for instance, they are used in the development of innovative scaffold topologies [32] and knotless suture anchors [33]. Thus, evaluating the mechanical characteristics of FFF materials is important at present.

Several investigations have been conducted on the mechanical characteristics and fatigue measurements of ABS and polycarbonate (PC) thermoplastics. Lee and Huang [34] employed a Tinius Olsen H50KS testing machine to determine fatigue data for various print orientations of ABS and ABS-plus materials produced by Stratasys FFF. In addition, Ziemian et al. [35] studied alternative construction orientations of ABS material to increase mechanical characteristics. Masood et al. [36] found that the tensile strength of FFF-processed polycarbonate (PC) was 70–75% when compared to molded and extruded PC components. Alhubail et al. [37] utilized the Taguchi approach to optimize the FFF processing conditions of ABS-M30i biomedical material and assess the surface roughness and tensile strength, resulting in upgraded component properties. One of the latest advancements involves the printing and recycling of low- and high-temperature thermoplastic composite materials. This innovative approach addresses sustainability concerns by repurposing recycled industrial polypropylene and glass-fiber waste into 3D-printing filament. Research by Sam-Daliri et al. [38] explores the recovery of particle-reinforced composite 3D-printing filaments from recycled materials, highlighting its potential for eco-friendly manufacturing practices. Additionally, Ritter et al. [39] have demonstrated the design and modification of a material extrusion 3D printer capable of producing functional gradient components using polyether ether ketone (PEEK). Their work showcases the versatility of FFF technology in fabricating complex and high-performance parts for various applications. These studies underscore the growing interest in and potential of FFF in sustainable manufacturing and advanced material processing.

The mechanical properties of plastic materials, especially fatigue characteristics, are widely recognized as being affected by the manufacturing procedures used in their production. Additive manufacturing, with its layer-by-layer construction technique, introduces anisotropic characteristics into the components that are produced, and the material properties of the constructed objects are influenced by the process conditions. Given the multitude of process variables that can influence the mechanical characteristics of FFF-produced materials, it is essential to comprehend how these properties differ among various polymers, processing techniques, and build orientations.

Afrose, M.F., et al. [40] investigated the fatigue properties of products made from PLA using FFF AM technology. Other studies have examined the impact of printing conditions on the mechanical characteristics (strength and modulus) of FDM ABS [41,42]. Enhancing the mechanical properties of Fused Deposition Modeling (FDM) polymers involves more than just optimizing 3D-printing conditions; reinforcement methods play a significant role. One common approach is the incorporation of fiber materials such as carbon, glass, Kevlar, and basalt into thermoplastic materials used in fused filament fabrication (FFF) or FDM. These fibers can be added either as short fibers or continuous fibers, providing varying degrees of reinforcement. Short fibers, for instance, have been shown to reduce delamination defects by creating bridges between adjacent printing layers. Several examples of FFF prototypes utilizing reinforced thermoplastic composites exist across different industries. For instance, a study on the mechanical performance of 3D-printed continuous-fiber Onyx composites for drone applications was conducted, showcasing the effectiveness of FFF technology in producing reinforced prototypes for various industrial applications [9]. Many inquiries have concentrated on enhancing the mechanical features of FDM polymers by optimizing 3D-printing conditions [43,44]. Nevertheless, few research works have concentrated on the fatigue performance of FDM ABS [16,45,46,47,48,49,50]. Safai and Shanmugam expounded upon the fatigue behavior of 3D-printed polymers by providing a detailed overview. Even so, former studies conducted on tension–tension or rotational bending fatigue testing on FFF ABS are available [16,50].

Several studies have been conducted to explore the effects of processing parameters on 3D-printed models [17,18,51,52,53,54,55]. Among the range of thermoplastic polymers used in FFF, ABS is the most utilized material due to its low cost, high strength, and temperature flexibility [56]. However, it is worth noting that the fatigue behavior of FFF-produced ABS has not been thoroughly explored in published studies. ABS is an environmentally friendly, compostable thermoplastic derived from renewable sources that boasts attractive appearance, good mechanical strength, low toxicity, and superior barrier properties, making it a suitable material for applications where cyclic-loading conditions may occur. Fatigue failure, which can occur in both metals and polymers, results from cyclic loading under yielding conditions, and it involves the initiation and propagation of fractures [16]. Therefore, when designing components for cyclic-loading applications, fatigue life should be regarded throughout the design process.

Based on the previous research, it has been suggested that FFF may offer better fatigue performance than traditional manufacturing methods for the final product [50,57]. Jap et al. [45] conducted a study on the impact of print directions and building orientations on the fatigue life of 3D-printed ABS samples. PLA is also a popular 3D-printing material with high mechanical strength; however, its ductility is lower than that of ABS [45]. Also, Eldeeb et al. [58,59] explored the influence of varying part orientations and build angles on the mechanical properties of 3D-printed PLA samples.

This study aims to bridge this knowledge gap by systematically investigating the influence of part-build directions (Upright, On Edge, Flat) and build orientation angles on the fatigue performance of ABS material processed using FFF. The research focuses on evaluating the number of cycles to failure under controlled cyclic tensile loading conditions for specimens fabricated with varying build parameters. Fractographic analysis of the fractured surfaces is conducted to gain insights into the failure mechanisms associated with different build orientations.

By elucidating the relationship between build parameters and fatigue behavior, this study provides valuable guidelines for optimizing the FFF process to enhance the fatigue resistance of ABS components. These insights are essential for designing durable and reliable 3D-printed parts for demanding applications where they are subjected to cyclic stresses.

## 2. Methodology, Experimental Details, and Sample Fabrication Conditions

In the present study, the ABS filament utilized as the raw material was produced by Bestfilament (Cologne, Germany), a reputable manufacturer in the field. The choice of white-colored filament was based on the rationale that the crack-growth paths in plastic zones would be more easily noticeable in this color. The ABS filament possessed a 1.75 mm diameter and a melting temperature of 230 °C.

The Picaso 3D-FFF printer’s type technique was utilized to produce the shaped fatigue samples in different printing directions (Upright, On Edge and Flat) and printing orientations ([0°, 90°], [15°, 75°], [30°, 60°], [45°]).

The Picaso 3D printers are FFF plastic jet printers that are supplied with 3D INC systems [26]. Features with an orientation of less than 45° in the printer bed do not require support structures for the sample’s inner features. This printer‘s bed and head move simultaneously in the X, Y, and Z directions. The polygon program utilized in this study converts the model’s STL file format into a printer file (PLG), which enables various printing fill densities, ranging from a solid model (100% fill density) to a hollow model (0% fill density), to be selected for generating a part.

For applications requiring high rigidity, solid models are preferable. Models with thin walls and air voids are well-suited for quick construction. The durable model, featuring intermediate wall thicknesses and internal voids, provides a compromise between the two. Samples were printed with a medium fill density of 50%.

The thermoplastics are melted by the printer head, resulting in a fine-flowing plastic material that sticks to the printer’s bed with a layer thickness of 0.25 mm. The printer’s table moves along the vertical axis (*Z*) as the printer head moves along the horizontal axes (X and Y directions), thereby enabling control over the shape and structure of the printed object. After each layer is printed, the build platform is lowered to allow for the deposition of the next layer. This process is repeated until the final layer is extruded, creating a complete 3D object.

**Figure 1:**
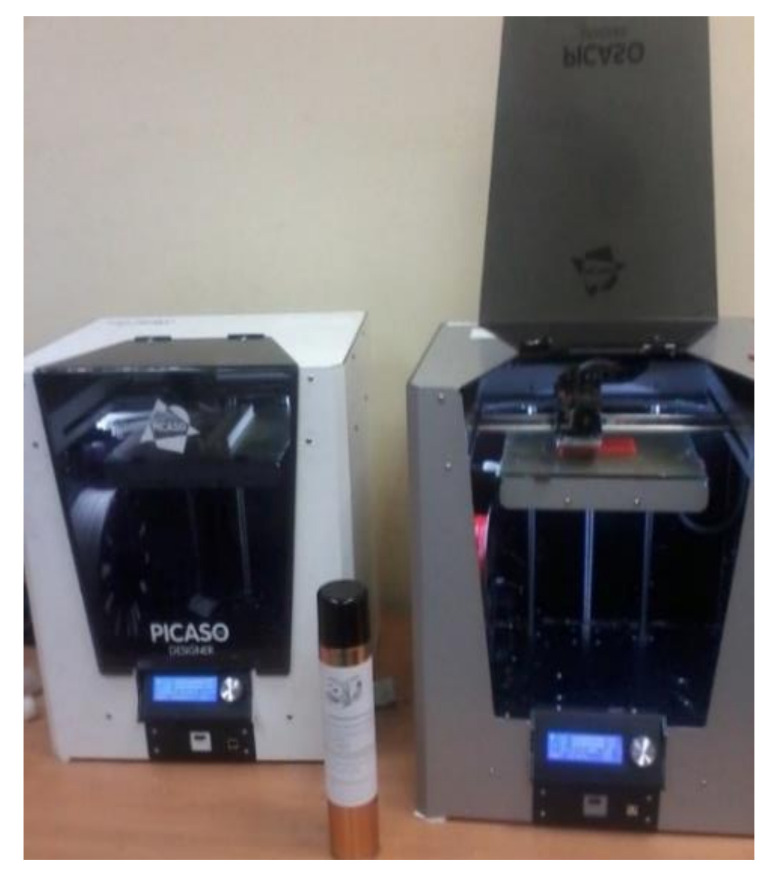
Overview of the Picaso 3D-printing machine.

A Picaso machine (intelligent additive manufacturing system), shown in Figure 1, was used for processing 1.75 mm of ABS. This FFF system has a build volume of 260 × 260 × 260 mm and is provided with both a heat-retaining bed and a build chamber. Fatigue test samples were printed directly on a preheated glass plate with the infill parameter set to 50% to create the samples. Table 1 presents the key printing parameters studied. On the build bed, all samples were developed in completely different directions with varying build orientation angles (XY surface). The 3D model was sliced into layers using the Polygon software.

### 2.1. The Standard for Sample Fabrication of Polymers for Uniaxial Fatigue

**Figure 2:**
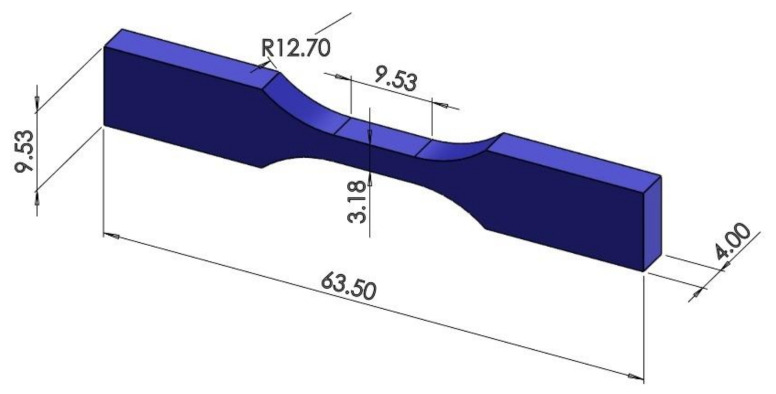
Dimensions of the sample according to ASTM Standard (D 638-TYPE IV) [60].

To investigate the fatigue behavior of the printed samples under tension, their geometry was determined according to ASTM D638 guidelines [60,61]. Figure 2 illustrates the dimensions of the sample and the specified directions and orientation angles necessary for placing the sample in the 3D printer. The 3D CAD model was generated with SolidWorks software (https://www.solidworks.com/) and then exported as a stereolithography (STL) file. We utilized Polygon software to generate the PLG file for the 3D printer. To achieve the required result, the indicated directions and orientation angles were generated as STL and PLG files for the printing process.

This research aims to assess how building direction and orientation affect the mechanical characteristics of 3D-printed ABS. Moreover, other aspects of the FFF process, including manufacturing time and cost, are also affected by these variables.

1. The building direction, which is defined by the plan printed on the table (X-Y plane) and the vertical direction (*Z*-axis), must be set by the operator during the placement of the samples on the 3D printer’s table.
2. The building orientation, on the other hand, refers to the route that the nozzle follows on each layer of the FFF part and can be adjusted through the 3D-printing G-Code.

To gain a more thorough grasp of the experimental results, the two 3D-printing conditions were carefully analyzed and set to encompass a reasonable range of printing parameters. Building direction and orientation angle were determined based on the default 3D profile, which not only ensured high-quality printing but also aligned with the standard setting ranges.

### 2.2. Build up Direction of the Samples

**Figure 3:**
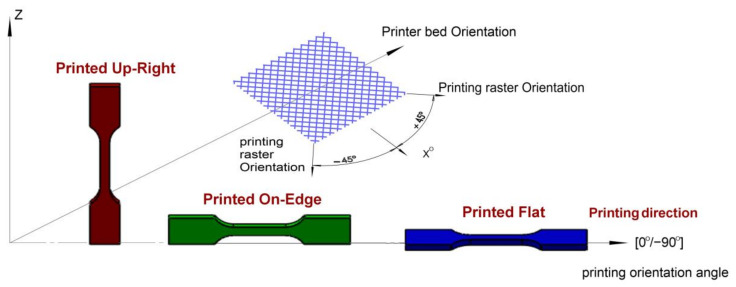
Three different building up directions (Upright, On Edge and Flat).

Building a part with different print directions and building orientation angles will often influence the product’s strength and mechanical characteristics. In this investigation, a 50% filling density mode, three build directions (Upright, On Edge and Flat) and different orientation angles ([0°, 90°], [15°, 75°], [30°, 60°], [45°]) were applied. Figure 3 demonstrates the three build directions for the ABS samples in the software application. The ABS samples were modeled in the software application, resulting in 63 samples of ABS materials in different orientations that were printed to conduct various tensile fatigue tests. Twenty-one samples were printed in each direction, with three samples in each orientation angle. The pattern of the extruded material will be affected by the positioning of the part on the build platform, as the printing head always moves horizontally (X and Y).

The positioning of a 3D model on the building area of an ABS FFF printer is a crucial factor that impacts the anisotropic behavior of printed parts. Significant differences in mechanical properties were observed along the printing directions: Upright, Flat, and On Edge (Figure 3). The investigation should indeed consider mechanical property variances for parts rotated at different printing angles to the reference system.

Figure 3 shows the notation of the positioning of a tensile sample. It was named according to the sample facing the printer table and the building-up direction. In this research, we propose using angles 0°, 15°, 30°, 45°, 60°, 75° and 90° for describing rotations in the printing planes. We can summarize the three distinct build directions in this investigation as follows:

- Upright: The longitudinal axis of the specimen (loading direction during testing) is aligned with the *Z*-axis of the printer, perpendicular to the build platform.
- On Edge: The longitudinal axis of the specimen is parallel to the build platform, with the shorter side (thickness) aligned with the *Z*-axis.
- Flat: The longitudinal axis of the specimen is parallel to the build platform, with the longer side (width) aligned with the *Z*-axis.

For each build direction, four different orientation angles were considered:

- [0°, 90°]: Alternating layers printed at 0° and 90° relative to the longitudinal axis.
- [15°, 75°]: Alternating layers printed at 15° and 75° relative to the longitudinal axis.
- [30°, 60°]: Alternating layers printed at 30° and 60° relative to the longitudinal axis.
- [45°]: All layers printed at 45° relative to the longitudinal axis.

### 2.3. Build up the Orientation of the Samples (Raster Angle)

**Figure 4:**
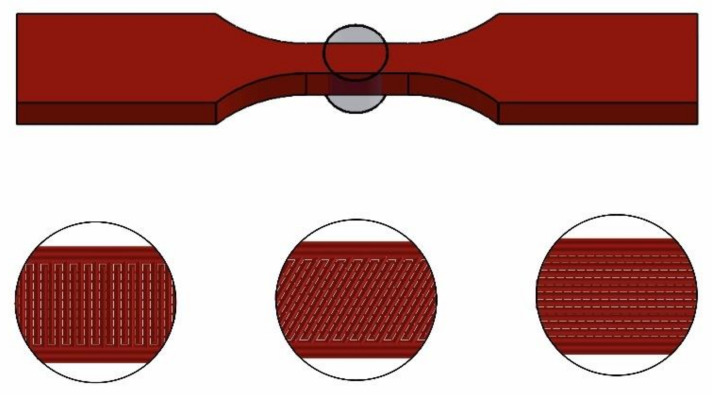
Raster angle.

The term “raster angle” refers to the orientation of the print paths relative to a fixed reference axis, which is a crucial parameter in understanding the anisotropic mechanical properties of 3D-printed materials. Figure 4 indicates a visual representation of the raster angle. This figure indicates the direction of the filament paths relative to the sample’s overall geometry.

The mechanical properties of 3D-printed parts are influenced by various raster conditions, as depicted in Figure 4, which presents the arrangement of a layer’s consecutive lines. The conditions include the raster angle, raster line width, raster line spacing, raster angle alternation between layers, number of wall lines, and distance between the raster and wall lines.

The mechanical behavior and fracture of 3D FFF-printed parts are significantly affected by the orientation of the raster lines, which can be of two types: unidirectional or alternating. To ensure clear and consistent notation, it is important to establish a standard convention for describing the alternating raster. To this end, we propose the use of θ1°/θ2° notation, where θ1° and θ2° denote the raster angles for two successive layers.

### 2.4. Sample Preparation

All tests were carried out on dog-bone-shaped samples made using a Picaso 3D-printer device in accordance with ASTM D638 [61], utilizing the Picaso 3D-printer machine. All test samples were successfully fabricated from ABS extruded from a 0.25 mm nozzle. The FFF machine control conditions remained at their default settings.

**Figure 5:**
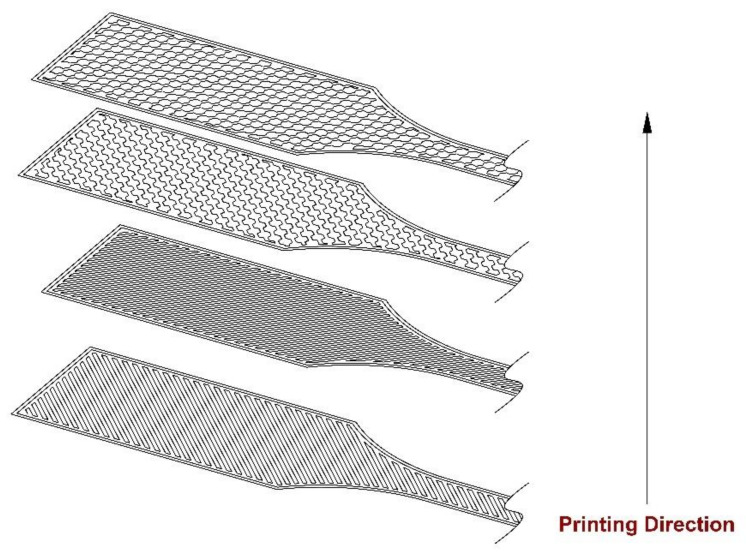
Construction of FFF samples; schematic indicates layering of four alternating orthogonal layers of bidirectional laminates.

Consider the case of Flat printing direction as an example. During fabrication, the sample direction was arranged in such a way that the minimum dimension of the component was parallel to the outward normal of the building table (i.e., the *Z*-axis of the machine), as illustrated in Figure 5. The thickness is 4 mm for each sample, which was made up of 16 layers. The final dimensions of the sample were 63.5 mm along the longitudinal *X*-axis and 9.53 mm along the *Y*-axis. The deposition methodology for each layer of a sample was determined by a raster angle or fiber orientation angle denoted as θ, with the measurement judged in reference to the +*X*-axis.

To conduct the strength study, samples were manufactured using two distinct mesostructures, which are combinations of filament orientations and layering patterns. The mesostructures were produced using two bidirectional laminates, consisting of 16 alternating orthogonal plies, as depicted in Figure 5. The unidirectional materials exhibited a fiber alignment angle of θ°= 0°, whereas the bidirectional laminates displayed alternating orthogonal plies of +θ°/−(θ°−90°) (specifically, +0°/−90°, +15°/−75°, +30°/−60°, +45°/−45°, +60°/−30°, +75°/−15°, and +90°/−0°). It can be considered that the two building orientations [+0°/−90°] and [+90°/−0°] have the same printing angle because of the repeatability of the filament orientation. Also, for the same reason, two building orientations—([+15°/−75°] and [+75°/−15°]) and ([+30°/−60°] and [+60°/−30°])—are the same printing angle, which leads to the same number of loading cycles.

A standard construction approach, which typically involves approximately up to 50% infill density and machine manufacturer-optimized conditions, was employed to minimize the manufacturing time while maintaining sufficient strength properties and dimensional stability. In our specific scenario, modified conditions were used, which included a raster orientation set at a 45-degree angle, diagonally crossing the layers along the longer dimension. This configuration was chosen to achieve a structure with optimal mechanical properties, potentially similar to the base material, as illustrated in Figure 5.

**Figure 6:**
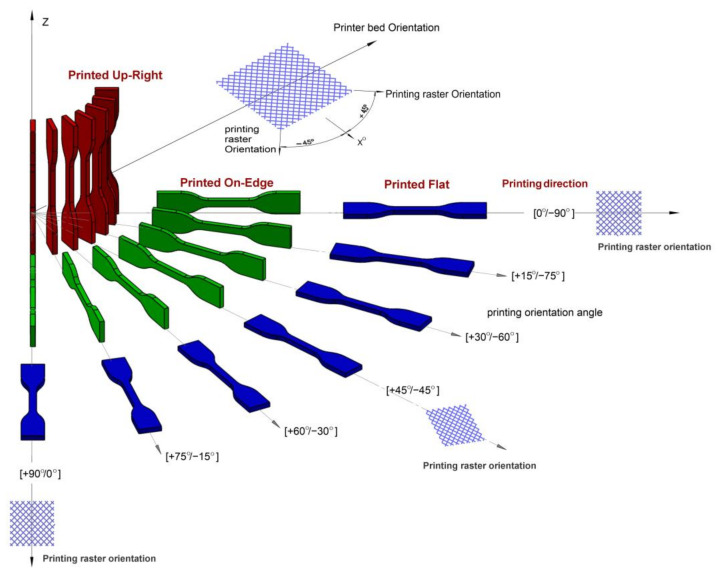
Production of samples for uniaxial tension–tension test with different building directions (Upright, On Edge and Flat) at different building orientation angles (0°, 15°, 30°, 45°, 60°, 75° and 90°).

Figure 6 provides an overview of the selected conditions for each parameter, which are illustrated and summarized. Figure 6 illustrates the production of samples for the uniaxial tension–tension test using different building directions (Upright, On Edge, and Flat) at various building orientation angles.

**Figure 7:**
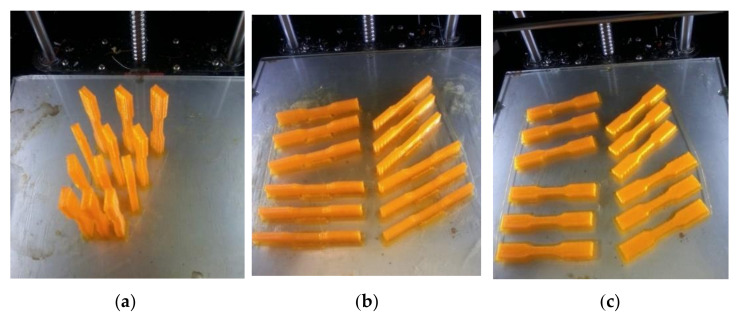
Samples arranged in the main three directions with different orientation angles. (**a**) Upright, (**b**) On Edge, (**c**) Flat.

Once a model is constructed, it must be converted into a sliced model, and G-code must be generated to guide the 3D printer head along a specific path. Numerous software options are available for this task. Polygon software version 2.0 was utilized as a slicer to transform the solid model into a sliced model with various printing parameters. Samples were positioned on the printer table in different orientations and angles. As shown in Figure 7, the samples are arranged in the main three directions at different angles. The G-code for the required passes will be generated automatically through the same program. Three samples were printed for each state in preparation for the fatigue test, with the number of loading cycles being calculated as the mean value of these three samples.

Table 2 outlines the tool path pattern of the deposited material for each of the three building directions employed in the tensile fatigue samples. The initial and final four layers were printed with raster angles of 45° and 135°. Conversely, the intermediate layers were printed using a honeycomb profile. The tool path remained consistent while the samples were positioned at different orientation angles.

### 2.5. Fatigue Testing

Fatigue tests are commonly utilized to assess the durability of components expected to undergo cyclic-loading conditions. In recent years, the fatigue behavior of polymeric materials has received greater attention, given their increasing use in major industries such as the aerospace, automotive, and biomedical fields. It is widely acknowledged that thermoplastic materials are particularly sensitive to various factors, including the loading cycle stress or strain amplitude, mean stress, stress or strain rate, initial flaws in the component, temperature, frequency, and environment. Various aspects must be regarded during the design process of a component intended for cyclic-loading conditions to ensure its desired fatigue life. By doing so, it is possible to gain a better understanding of how to select appropriate materials for specific applications.

**Figure 8:**
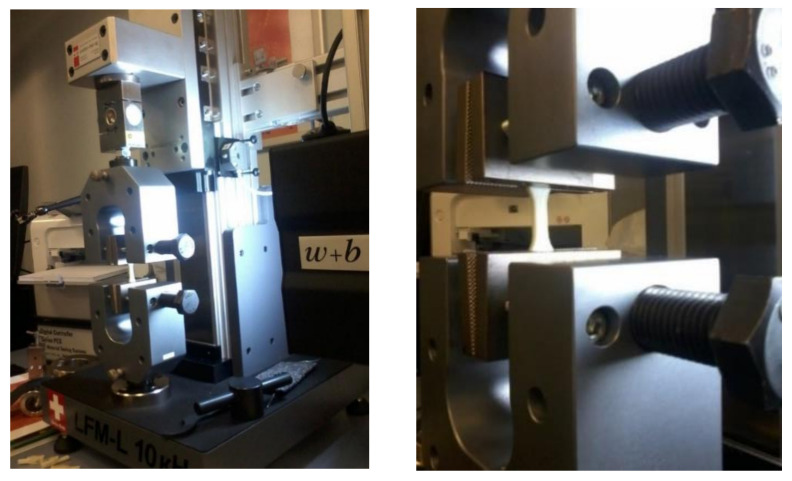
Sample holding in a 10 KN Walter + Bai LFM-L testing machine: (**a**) general view of the test machine, (**b**) installation of the sample in the wedge clamp of the machine.

A Walter + Bai LFM-L universal testing machine (Löhningen, Switzerland) of a new version with a maximum load capacity of 10 KN was utilized for fatigue testing. The fatigue tests were conducted on the samples using this testing machine, and the technical specifications of the machine used in this study are presented in Table 3. To monitor and record all test results, testXpert_ II intelligent software was utilized to manage the machine. It was found that increasing the frequency raises the core temperature of the sample, which leads to reduced fatigue life by allowing material flow and enhancing ductility, resulting in highly concentrated deformation at the weakest segment of the gauge length. In contrast, lower frequency results in longer fatigue life, primarily due to brittle failure with limited deformation over the sample’s gauge length [61]. As a result, the tests were conducted at a frequency of 1 Hz at room temperature. Wedge-style cross-hatched grips were utilized to ensure decent sample gripping, as shown in Figure 8, since no sample temperature control device was provided during the test, in accordance with the test program’s criteria.

The main condition of the test is the frequency. The frequency of the cyclic loading is 1 Hz. The test results presented several loading cycles until fracture. For tension–tension tests, it is common to apply cyclic loading that varies between a minimum and a maximum stress value. At least three specimens were tested for each combination of build direction and orientation angle.

**Figure 9:**
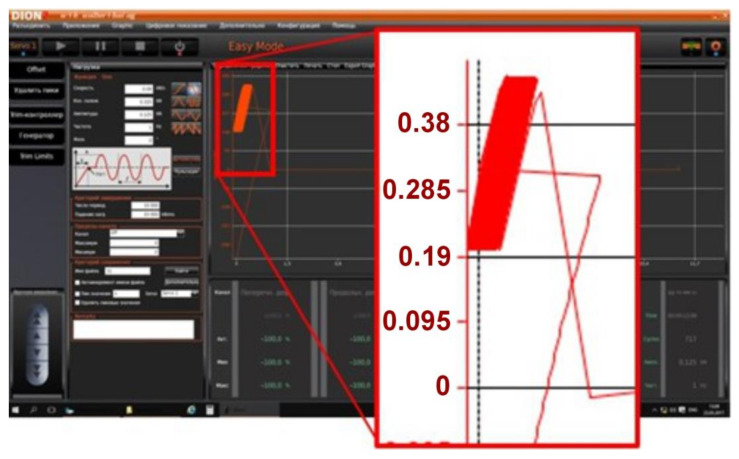
Graphical user interface (test sample no. 49); the test results presented in several loading cycles until fracture.

Stress or Strain Amplitudes and Stress Ratios: The fatigue tests were conducted under tension–tension conditions with a sinusoidal loading profile (Figure 9). A sinusoidal profile was chosen as it represents a common type of cyclic loading experienced by components in real-world applications.

The tests were performed with a constant stress ratio (R) of 0.1. This means the minimum stress applied during each cycle was 10% of the maximum stress, maintaining a tensile load throughout the test.

Uniaxial Tensile Test Results: Table 4 includes the results of the preliminary uniaxial tensile tests. This table summarizes the key tensile properties (e.g., yield strength, ultimate tensile strength, elongation at break) for each build direction and orientation.

The specific maximum stress level for each test specimen was determined from preliminary uniaxial tensile tests (results summarized in Table 4). We targeted maximum stress levels that would induce fatigue failure within a practical number of cycles for each build orientation. This approach allowed us to observe clear differences in fatigue life between the various printing conditions.

The impact of different construction angles and part orientations on the mechanical characteristics of 3D-printed PLA samples was thoroughly investigated by Eldeeb et al. [58,59]. The detailed steps of the uniaxial tensile tests are demonstrated in this investigation.

Uniaxial tensile tests were performed according to ASTM D638-14 [60] using a Walter + Bai LFM-L universal testing machine with a nominal force of 10 kN. The specimens were loaded at a constant crosshead speed of 0.4 mm/s, equivalent to a strain rate of approximately 8%/min, ensuring a quasi-static loading condition. An initial grip separation of 50 mm was used to accommodate the specimen geometry and allow for sufficient elongation before fracture. Strain was measured using a 25 mm gauge length extensometer attached to the specimen. Load and displacement data were acquired frequently, ensuring adequate data resolution for determining tensile properties. Specimens were conditioned at 23 ± 2 °C.

Stress–Number of Cycles (S–N) Curves: Obtaining the value of presenting S–N curves was the main research target in this investigation. However, this study focused on comparing the fatigue life (number of cycles to failure) at a single, predetermined stress level for each build orientation. This approach allowed us to efficiently investigate the influence of build direction and orientation on fatigue performance without generating full S–N curves for each condition, which would have significantly increased the experimental effort.

Figure 8 represents a general view of the Walter + testing machine Bai LFM-L with a nominal force of 10 kN (Figure 8a), according to a symmetrical loading cycle. Sample installation in the wedge clamp of the machine is illustrated in Figure 8b.

Figure 9 presents the loading conditions (dynamic test settings) used to control the tension–tension mode fatigue testing process. These conditions include frequency, amplitude, and load. Figure 9 also presents the graphical user interface when conducting an experiment (test sample no. 49), and the test results are presented in the number of loading cycles until fracture.

**Figure 10:**
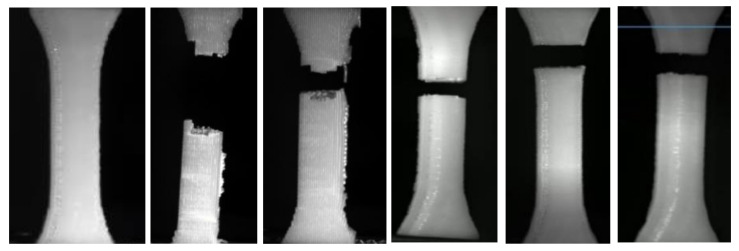
Some samples before and after destruction; the crack formation is shifted from the central part towards the sample clamps, as shown in the sample failure examples.

Under cyclic loading, parts generally fail in high-stress concentration areas. In some cases, failures can occur in the middle of a homogeneous part. Figure 10 illustrates the failure pattern of tested samples in three different build directions, namely On Edge, Upright, and Flat. Due to the different build patterns in each orientation, the failure pattern of the test samples appeared in different positions, which affects the mechanical properties of the material.

The test samples consistently failed at the same location along the neck due to the build pattern routes aligning with the length of the samples, as illustrated in Figure 10. These routes likely created stress concentrations, leading to fatigue failure. By modifying the printing orientation and angle, failure occurred in different areas—where the components experienced high stress.

It can be seen that the destruction of samples occurs closer to the capture area, which is undesirable (Figure 10). There are also distinctly discernible differences in the formed places of destruction (smooth cut and destruction with protrusions). This suggests that the internal structure has an impact on the destruction. The direction of 3D printing in the plane of the table (XOY) also has an impact.

## 3. Results

This study aims to evaluate the effects of building direction and orientation angle on the fatigue endurance of 3D-printed components manufactured using FFF. The target material used in the investigation is ABS, which is commonly utilized in 3D printing. These samples with different printing orientation angles are tested to determine the best printing direction and discover which one performs better in the S vs. Nf curve.

### 3.1. Numerical Assessment

As previously mentioned, the samples’ fatigue testing involved subjecting them to uniaxial fatigue in tension–tension mode. The maximum and minimum loads were controlled for each sample during the pullout and retraction process. Table 5 shows the number of loading cycles of the samples fabricated using three different printing directions and various printing orientation angles. It was found that the fatigue life of the samples was influenced by the construction styles and orientations, rather than the material properties. Each experiment was printed and tested three times, and the value in the table represents the mean of the three samples for each condition.

The fatigue-test-output Excel document includes tables for the maximum number of cycles. The data were processed using Excel software (Microsoft office 365) to obtain the number of cycles for each sample. Table 5 displays the average values of the number of cycles. These values were arranged according to sample direction (Upright, On Edge and Flat). In each direction the samples were arranged in different orientations with different angles ([0°, 90°], [15°, 75°], [30°, 60°], [45°]). The results indicate that the maximum number of cycles is obtained in the On-Edge direction.

**Figure 11:**
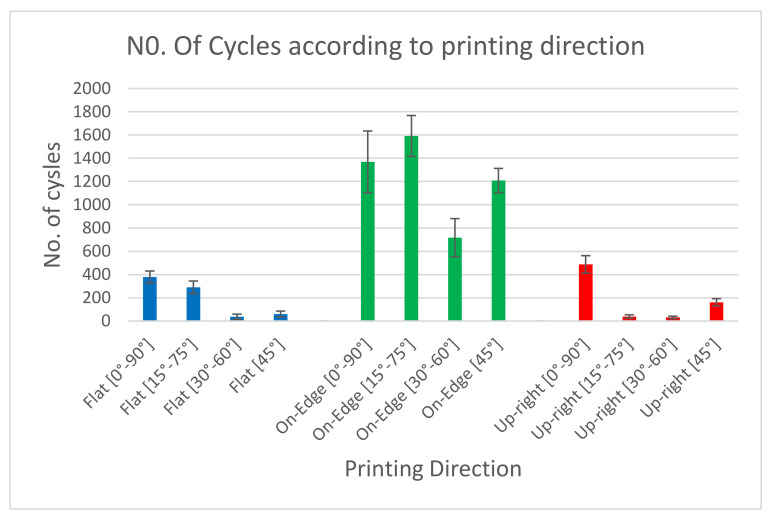
Bar chart of endurance of samples according to printing direction.

Figure 11 illustrates the number of cycles for samples in the three directions, showing that ABS components built in the On-Edge direction exhibit higher cycle softening than those built in the Flat and Upright directions. The cyclic deformation behavior varies depending on the loading direction and type of material, with cyclic hardening and cyclic softening occurring due to the misplacement of the substructure inside the material. The cyclic deformation behavior is more pronounced at the beginning of the cyclic loading, and the material gradually stabilizes as the cycling continues.

**Figure 12:**
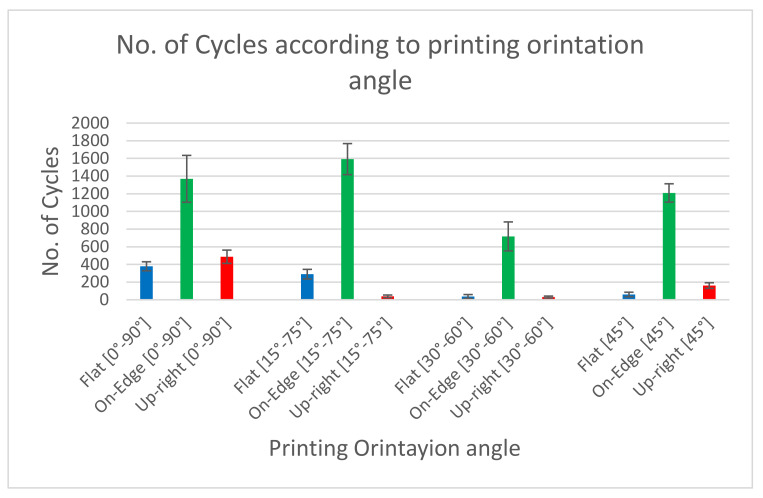
Bar chart of endurance of samples according to the printing orientation angle.

Table 6 shows the average values of the number of cycles. These values were arranged according to printing orientation angle ([0°, 90°], [15°, 75°], [30°, 60°], [45°]). At each angle, the samples were arranged in different printing directions (Upright, On Edge and Flat). It is noticed that the number of cycles obtained during fatigue testing in the [0°,90°] printing angle is the maximum number of cycles. Figure 12 shows the number of cycles for the samples of four different angles named [0°, 90°], [15°, 75°], [30°, 60°], and [45°]. It is noteworthy that the cyclic deformation behavior of the samples is influenced by the printing orientation angle. Based on the information presented in Table 6, it is possible to conclude that the Upright- and Flat-type build orientations result in significantly lower mechanical behavior than the On-Edge configurations. The interlayer breakage in the test samples causes this mechanical behavior.

In order to appropriately evaluate fatigue-behavior polymeric components, it is crucial to conduct fatigue tests under conditions that closely resemble those of the service environment. The S–N approach, which involves plotting the number of cycles to failure against the applied stress level, is commonly accepted in the research community for assessing the effects of cyclic loading on materials.

### 3.2. Fractographic Assessment

Following fatigue failure, the fracture surfaces of representative samples were examined using optical microscopy to analyze the fracture morphology and identify potential failure mechanisms.

Upon analyzing the fracture surfaces of the samples that underwent tensile fatigue tests, it was observed that the prevalence of various damage modes varied based on the mesostructure. All the test samples had some degree of microscopic cracks, indicating brittle fracture.

**Figure 13:**
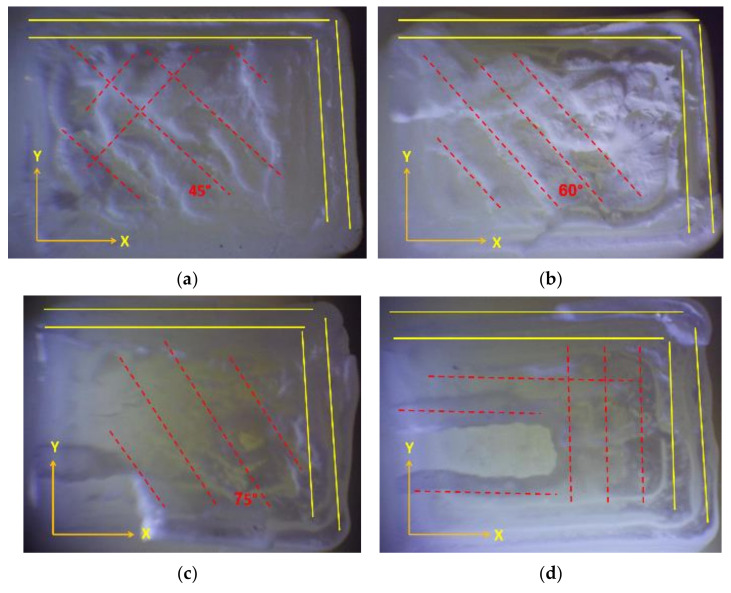
Optical images of fracture surfaces from tensile fatigue tests of Upright samples printed with different orientation angles (**a**) [0°, 90°], (**b**) [15°, 75°], (**c**) [30°, 60°], (**d**) [+45°/−45°].

**Figure 14:**
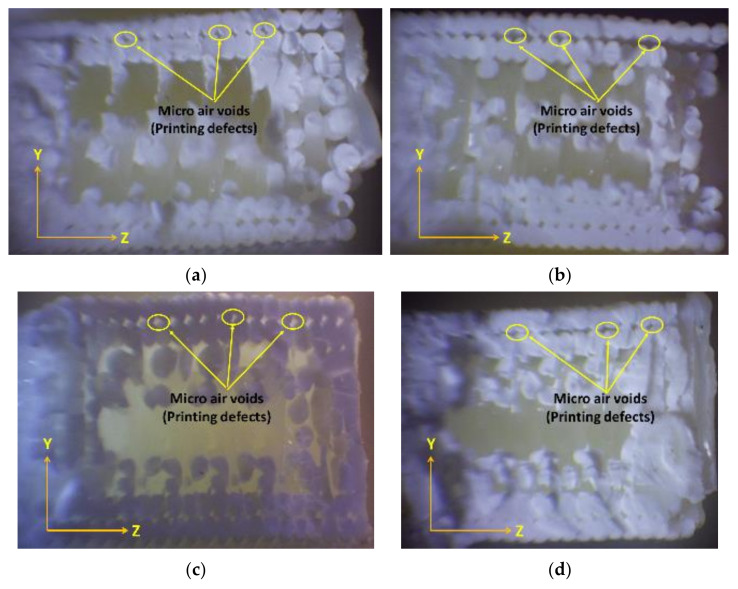
Optical images of fracture surfaces from tensile fatigue tests of On-Edge samples printed with different orientation angles (**a**) [0°, 90°], (**b**) [15°, 75°], (**c**) [30°, 60°], (**d**) [+45°/−45°].

The damage modes observed in the tested samples were dominated by filament crazing and cracking at the mesostructure level, particularly in the On-Edge and Flat printing directions. This led to an uneven external appearance of the fracture surface due to inconsistent crack paths and variable lengths of the fractured fibers, as depicted in Figure 13 and Figure 14. The yellow lines highlight the boundary layout of the printed sample, while the red dotted lines emphasize the printing angles within the printing layout.

The failure modes were affected by the strength of the bonds and the size of the air voids, which differed between the samples.

The FFF process, which uses a continuous filament, inevitably includes some level of internal flaws, such as voids and approximations at curved areas. This trade-off between geometric precision and bond strength is widely recognized.

The samples to be studied were printed with defined conditions at various building directions and building orientation angles, with a wide variety of cycle life at failure for each stress level. Noticing the high-magnification fracture surfaces of the test samples, some observations could be produced.

**Figure 15:**
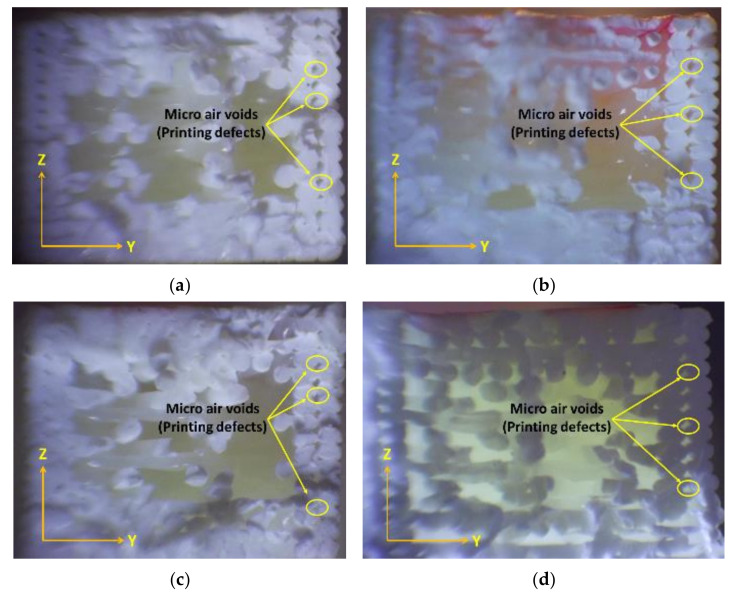
Optical images of fracture surfaces from tensile fatigue tests of Flat samples printed with different orientation angles (**a**) [0°, 90°], (**b**) [15°, 75°], (**c**) [30°, 60°], (**d**) [+45°/−45°].

A closer examination of the test samples that demonstrated the highest endurance in each test, conducted with varying building directions and orientation angles, reveals a more accurate deposition temperature and filament placement. This is evident when analyzing Figure 13, Figure 14 and Figure 15 (Upright, On Edge, and Flat), as it is completely obvious that the fracture surface is almost acquired, allowing us to make a distinction between the filament’s cross-sections.

The fracture in the upper part of the samples printed with the On-Edge printing direction demonstrates the beginnings of beach lines, which is indicative of the impact of fatigue tests. Beach lines can be seen in the ABS printed material because they exhibit different damage mechanisms with various deposition raster angles.

The experimental findings align with previous research, with the results positioned at the upper end of the average. Nonetheless, direct comparison is difficult due to disparities in material type, manufacturing conditions, and test sample types. Nevertheless, our results offer valuable insights into critical variables such as the influence of constant stress on alternating components.

Fractographic analysis of the fractured surfaces revealed distinct failure modes depending on the build direction and orientation angle:

Upright: Specimens printed in the Upright direction predominantly exhibited interlayer failure, characterized by crack propagation along the layer interfaces. This failure mode is attributed to the weak bonding strength between adjacent layers, exacerbated by the stress concentration at the layer boundaries when the load is applied perpendicular to the layer planes.

On Edge: Specimens fabricated in the On-Edge direction, particularly those with [15°, 75°] and [45°] orientations, displayed a combination of transgranular and intergranular fracture modes. The presence of transgranular fracture, where cracks propagate through the filament rather than along the boundaries, suggests improved interlayer bonding and load transfer compared to the Upright direction.

Flat: Specimens printed in the Flat direction showed a mixed-mode fracture behavior, with evidence of both interlayer and intralayer failure. The fracture surfaces appeared rougher compared to the On-Edge specimens, indicating a more tortuous crack path.

## 4. Discussion

Fused filament fabrication is a technique that has been extensively utilized across a diverse range of fields, as well as in industrial production, research and development, and home-based usage. ABS is a resource material that can be used to produce industrial products if the mechanical and physical properties are predictable and repeatable during the manufacturing process.

The mechanical performance of FFF ABS components is influenced by various factors, such as filament production, geometric configuration, process variables, 3D-printing apparatus, post-production treatment, and mechanical assessment protocols. In this study, the build-up directions and printing raster angle orientations have been investigated with a focus on the process variables that have garnered the most attention in the literature. It is crucial to establish suitable test samples for conducting mechanical evaluations of FFF products, as evidenced by a substantial quantity of fatigue samples. In the absence of detailed regulations, testing a greater number of samples may be considered to reduce errors resulting from failure outside the adjusted area.

The findings demonstrate that vertically printed samples exhibit substantially inferior mechanical attributes compared to Flat and On-Edge printed samples, which are influenced by the sample orientation relative to the build plate direction. In contrast to unidirectional rasters, alternating rasters exhibit superior mechanical features. The anisotropic behavior of FFF ABS components is significantly associated with conditions such as raster angle, build orientation, and failure mode, namely interlayer, inter-line, and in-layer/in-line failure.

Previous experiments focused on examining the mechanical properties of samples manufactured with unidirectional and alternating rasters. It was observed that samples with alternating rasters displayed superior mechanical characteristics. To comprehensively evaluate the mechanical behavior, the raster angle should be analyzed as a function that is closely linked to the sample’s build orientation.

The failure of fatigue samples can be impacted by the printing building directions and build orientations. Three distinct types of failure have been identified, namely interlayer failure, inter-line failure, and in-layer (or in-line) failure.

- Interlayer failure occurs when a fracture forms between two adjacent parallel layers, as observed in tensile test samples with build-up printing orientations.
- Inter-line failure happens when the cracking and breaking surface is collinear with the printing raster angle.
- In contrast, in-layer or in-line failure takes place when the fracture surface is misaligned with either the raster angle or the interface between two neighboring parallel layers.

Inter-line failure is related to a reduction in diffusion between raster lines. The presence of inter-line failure in the Flat printing directions in samples with a unidirectional raster angle of θ = 90° is the reason for their lower tensile strength relevant to samples with θ = 0° or θ = 45°. The raster arrangement can intensify stress concentration in the radius region of the tensile samples, specifically regarding a unidirectional raster.

To enhance FFF technology, it is essential to prioritize the processing conditions that have the greatest impact on the mechanical characteristics of the printed model. For test samples printed in various directions and orientations, the following process parameters were found to have the most significant influence on ultimate tensile strength: infill density, infill orientation, layer thickness, head temperature, presence of a contoured wall, wall thickness, and printing speed.

Regarding the specific results for the On-Edge building orientation, our findings indicate that samples printed in this orientation exhibited the longest duration of resistance to fatigue forces (tension–tension mode) before fracturing. This superior performance can be attributed to several key factors. In the On-Edge orientation, the layers are aligned perpendicular to the primary direction of the load during fatigue testing. This alignment allows the load to be distributed more evenly across the layers, reducing the stress concentration at any single point and enhancing the overall structural integrity. The On-Edge orientation tends to improve interlayer bonding, which enhances the fusion between layers. Stronger bonding at the interfaces contributes to higher resistance. Conversely, in the Flat orientation, the layers are parallel to the loading direction. This setup tends to concentrate stress at the interfaces between layers, which are the weakest points due to potential gaps or less-than-optimal fusion between layers. The concentration of stress facilitates the initiation and propagation of cracks along these interfaces, leading to premature failures under cyclic-loading conditions.

The significant variations in fatigue life observed for ABS specimens fabricated with different build directions and orientations underscore the importance of considering these parameters during the design and fabrication of FFF-printed components subjected to cyclic loading.

### 4.1. Influence of Build Direction on Fatigue Performance

The superior fatigue resistance of specimens printed in the On-Edge direction can be attributed to several factors:

- Improved Load Transfer: In the On-Edge orientation, the layers are aligned perpendicular to the primary tensile loading direction. This arrangement allows for more efficient load transfer across the layers, reducing stress concentrations at the layer interfaces, which are inherently weaker than the bulk material.
- Enhanced Interlayer Bonding: The On-Edge build direction typically results in better fusion between adjacent layers compared to the Upright direction. This improved interlayer bonding enhances the overall structural integrity and resistance to crack initiation and propagation.
- Reduced Void Formation: The On-Edge orientation can minimize the formation of voids during the printing process. Voids act as stress concentrators and can significantly reduce fatigue life.

Conversely, the Upright build direction, where the layers are stacked parallel to the loading direction, leads to poor load transfer and increased stress concentrations at the layer interfaces, resulting in premature fatigue failure.

### 4.2. Effect of Orientation Angle on Fatigue Behavior

Within each build direction, the orientation angle also plays a crucial role in influencing fatigue life. The [15°, 75°] and [45°] orientations consistently exhibited better fatigue performance compared to the [0°, 90°] and [30°, 60°] orientations. This observation can be attributed to the following:

- Stress Distribution: The angled orientations promote a more even distribution of stresses throughout the material, reducing localized stress concentrations that can initiate fatigue cracks.
- Crack Deflection: When a crack initiates in an angled layer, it encounters an interface at an angle, forcing it to deviate from its path. This crack-deflection mechanism increases the energy required for crack propagation, delaying fatigue failure.

### 4.3. Fractographic Evidence and Failure Mechanisms

The fractographic observations corroborate the fatigue life results and provide further insights into the failure mechanisms. The predominance of interlayer failure in Upright specimens highlights the weak link in this build direction. The presence of transgranular fracture in On-Edge specimens, especially those with angled orientations, confirms the improved interlayer bonding and more effective load transfer. The mixed-mode failure in Flat specimens suggests a complex interplay of factors influencing crack initiation and propagation.

## 5. Conclusions

This investigation provides a comprehensive understanding of how build direction and orientation angles influence the fatigue performance of ABS components fabricated using FFF technology. We conducted fatigue tests under tension–tension loading on specimens printed with varying build parameters, specifically focusing on a 50% infill density. The results offer crucial insights for designers and manufacturers seeking to optimize the FFF process and produce durable ABS parts for applications involving cyclic loading.

Our key finding is that the On-Edge build direction, where layers are printed perpendicular to the primary loading direction, consistently demonstrated superior fatigue resistance compared to the Upright and Flat orientations. This superior performance is attributed to several key factors: enhanced load transfer across layers, improved interlayer bonding, and reduced void formation. Notably, the [15°, 75°] orientation angle within the On-Edge build direction yielded the highest fatigue life, highlighting the synergistic effect of combining optimized build direction and angled layer orientations. This advantage is attributed to their ability to promote a more even distribution of stress throughout the material and enhance crack-deflection mechanisms, thereby delaying crack initiation and propagation.

Fractographic analysis of the fractured surfaces supported these findings, revealing distinct failure modes associated with different build orientations. Upright specimens predominantly exhibited interlayer failure, highlighting the weakness of interlayer bonds in this orientation. In contrast, On-Edge specimens, particularly those with angled orientations, showed evidence of transgranular fracture, confirming improved interlayer bonding and more effective load transfer.

These results underscore the significant impact of build direction and orientation angle on the fatigue behavior of FFF-printed ABS components. By strategically choosing the On-Edge build direction and incorporating angled layer orientations, designers and manufacturers can significantly enhance the fatigue resistance and overall durability of these 3D-printed parts. This knowledge is essential for creating reliable ABS components for demanding applications where cyclic loading is expected.

Further research is warranted to explore the influence of other printing parameters, such as infill density, layer thickness, and printing speed, on the fatigue performance of FFF-printed ABS. Investigating the material’s fatigue behavior under varying strain rates, temperatures, and environmental conditions would also provide valuable insights for practical applications.

## Figures and Tables

**Table 1:** Selected conditions for 3D printing of ABS samples [58,59].

Printing Conditions	Value
Nozzle temperature (°C)	230
Bed temperature (°C)	110
Chamber temperature (°C)	30
Layer height (mm)	0.25
Printing speed(mm/s)	45
Infill density (%)	50%
Infill pattern	Honeycomb
Shell	Wall line Count
Shell thickness (mm)	0.5
Number of bottom/top layers	2/2
Number of contours (wall)	2
Filament diameter (mm)	1.75
Infill line directions (relative to the long axis of the test bar) (°)	(45/−45)

**Table 2:** Printing tool-path material deposition.

Angle	Upright First and Third Layers	Upright Second and Fourth Layers	Upright Fifth Layer	Upright Sixth Layer
Angle [0°, 90°]	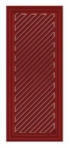	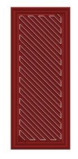	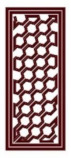	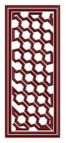
Angle [15°, 75°]	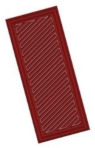	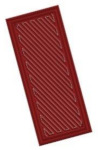	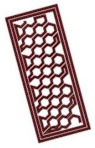	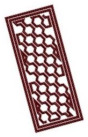
Angle [30°, 60°]	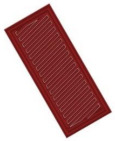	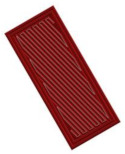	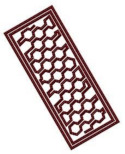	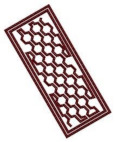
Angle [45°]	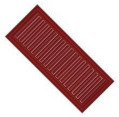	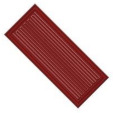	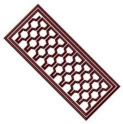	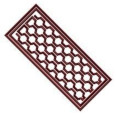

**Table 3:** Technical data of Walter + Bai LFM-L 10 kN test machine.

Maximum load (KN)	10
The distance between clamps (mm)	0–1183 mm
Accuracy standards from 0.2 to 10 MN tensile/compression	ISO 7500-T2
Stroke (mm)	1000
Noise level (dB)	50 dB
Dimensions W × D × H (mm)	350 × 430 × 1630
Weight (kg)	90
Power supply	230 V, 50 Hz, 16 A

**Table 4:** Summary of uniaxial tensile test results for ABS specimens.

Building Direction	Orientation Angle	Yield Strength (MPa)	Ultimate Tensile Strength (MPa)	Elongation at Break (%)
Upright	[0°, 90°]	35.2	42.5	8.3
	[15°, 75°]	33.8	40.1	7.6
	[30°, 60°]	34.5	41.2	7.9
	[45°]	32.9	39.5	7.2
On Edge	[0°, 90°]	41.7	48.3	10.2
	[15°, 75°]	43.2	50.1	11.5
	[30°, 60°]	40.5	47.1	9.8
	[45°]	42.3	49.2	10.8
Flat	[0°, 90°]	38.9	45.6	9.1
	[15°, 75°]	37.5	44.2	8.7
	[30°, 60°]	38.2	44.9	8.9
	[45°]	36.8	43.5	8.5

**Table 5:** Average fatigue number of loading cycles, grouped according to printing direction.

No	Material	Filling Percentage (%)	Orientation Angle	Printing Direction	No of Loading Cycles (Mean Value)	Standard Deviation
1, 2, 3, 19, 20, 21	ABS	50%	[0°–90°]	Flat	379	50.9
4, 5, 6, 16, 17, 18	ABS	50%	[15°–75°]	Flat	290	54.2
7, 8, 9, 13, 14, 15	ABS	50%	[30°–60°]	Flat	37	22.1
10, 11, 12	ABS	50%	[45°]	Flat	60	25.5
22, 23, 24, 40, 41, 42	ABS	50%	[0°–90°]	On-Edge	1369	265.4
25, 26, 27, 37, 38, 39	ABS	50%	[15°–75°]	On-Edge	1592	175.8
28, 29, 30, 34.35, 36	ABS	50%	[30°–60°]	On-Edge	717	164.1
31, 32, 33	ABS	50%	[45°]	On-Edge	1209	103.8
43, 44, 45, 61, 62, 63	ABS	50%	[0°–90°]	Upright	487	75.2
46, 47, 48, 58, 59, 60	ABS	50%	[15°–75°]	Upright	39	14.7
49, 50, 51, 55, 56, 57	ABS	50%	[30°–60°]	Upright	31	10.6
52, 53, 54	ABS	50%	[45°]	Upright	161	31.5

**Table 6:** Average fatigue number of loading cycles, grouped according to orientation angle.

No	Material	Filling Percentage (%)	Orientation Angle	Printing Direction	No of Loading Cycles (Mean Value)	Standard Deviation
1, 2, 3, 19, 20, 21	ABS	50%	[0°–90°]	Flat	379	50.9
22.23.24, 40, 41, 42	ABS	50%	[0°–90°]	On-Edge	1369	265.4
43, 44, 45, 61, 62, 63	ABS	50%	[0°–90°]	Upright	487	75.2
4, 5, 6, 16, 17, 18	ABS	50%	[15°–75°]	Flat	290	54.2
25.26.27, 37, 38, 39	ABS	50%	[15°–75°]	On-Edge	1592	175.8
46, 47, 48, 58, 59, 60	ABS	50%	[15°–75°]	Upright	39	14.7
7, 8, 9, 13, 14, 15	ABS	50%	[30°–60°]	Flat	37	22.1
28, 29, 30, 34, 35, 36	ABS	50%	[30°–60°]	On-Edge	717	164.1
49, 50, 51, 55, 56, 57	ABS	50%	[30°–60°]	Upright	31	10.6
10, 11, 12	ABS	50%	[45°]	Flat	60	25.5
31, 32, 33	ABS	50%	[45°]	On-Edge	1209	103.8
52, 53, 54	ABS	50%	[45°]	Upright	161	31.5

## Data Availability

The original contributions presented in the study are included in the article, further inquiries can be directed to the corresponding authors.
